# Organic Vegetable Chips: Exploring Romanian Consumers’ Preferences in Relation to Socio-Demographic Factors

**DOI:** 10.3390/foods12183374

**Published:** 2023-09-08

**Authors:** Andreea Barbu, Violeta Alexandra Ion, Mihai Frîncu, Nicoleta Defta, Viorica Lagunovschi-Luchian, Liliana Aurelia Bădulescu

**Affiliations:** 1 Research Center for Studies of Food Quality and Agricultural Products, University of Agronomic Sciences and Veterinary Medicine of Bucharest, 59 Marasti Blvd., 011464 Bucharest, Romania; andreea.stan@qlab.usamv.ro (A.B.); mihai.frincu@qlab.usamv.ro (M.F.); liliana.badulescu@qlab.usamv.ro (L.A.B.); 2 Faculty of Animal Productions Engineering and Management, University of Agronomic Sciences and Veterinary Medicine of Bucharest, 59 Marasti Blvd., 011464 Bucharest, Romania; 3 Faculty of Horticulture, University of Agronomic Sciences and Veterinary Medicine of Bucharest, 59 Marasti Blvd., 011464 Bucharest, Romania; vluchian@hotmail.com

**Keywords:** organic vegetable, chips preference, dried vegetables, respondents’ profile, consuming frequency

## Abstract

In recent years, people have become increasingly interested in adopting a healthy diet, which also extends to healthy snacks, such as chips. Understanding the interplay of factors that influence the preference decisions concerning food products is very helpful in market segmentation for identifying specific groups of consumers with similar needs. This study aims to obtain a better understanding of Romanian consumers’ preference for organic vegetable chips vs. classic potato chips. The research involved a transversal, cross-sectional, descriptive, exploratory, and correlational design. Data were collected based on a questionnaire (1060 participants) and processed with the SPSS 22 program, using the Pearson chi-square test and binary logistic regression as statistical procedures. Significant differences were found regarding the distribution of the respondents who prefer organic vegetable chips vs. classic potato chips based on socio-demographic characteristics. The results of the binary logistic regression analysis (χ^2^ = 102,906, df = 22, N = 909, *p* < 0.001) indicate that education level and frequency of consumption have a statistically significant impact on the preference for organic vegetable chips. The obtained results can contribute to a better understanding of Romanian consumers’ preferences, acting as a knowledge stage in the adoption of a healthy eating style.

## 1. Introduction

The world’s population exceeds 8 billion people, and, as Lelieveld noted in 2012, we all want to be happy, have enough to eat, have families, and prosper [1]. The modern consumer has become more attentive to their health and sustainable consumption [2], and their attitudes and beliefs are factors that influence the acceptance or rejection of new foods [3,4]. Understanding the complexity and determinants of food choices is important for consumers, producers, and retailers.

One of the most applied models in the consumer behavior approach is based on the Theory of Planned Behavior (TPB), which is also used to predict the intention and actual behavior of a consumer regarding the purchase of commodities [5,6,7,8]. One study [7] applied the TPB to understand the behavioral intentions in choosing healthy foods, purchasing snacks, or frequently purchasing classic potato chips, which have regained research interest due to global changes [9,10,11].

Researchers, such as Tomić Maksan and Jelić (2022) [6], consider attitude to be the strongest predictor, meaning that consumers who perceived the purchase of classic chips as important, enjoyable, and fun, as well as associated it with positive emotions, also had a stronger intention to buy them.

A buying habit is defined as a behavior that is performed automatically and without the individual’s awareness [6,12], while attitude is a favorable or unfavorable evaluation of a certain outcome (e.g., consuming fast food). Favorable attitudes support behavioral intention, while negative attitudes hinder it [7]. Buying habits related to certain food products have been studied by some authors in the form of purchase frequency [6]. Attitudes have been studied concerning the food products evaluated as healthy/unhealthy and pleasant/unpleasant [13].

The shift towards healthy consumption practices encourages the development of products that correspond to sustainable diets, defined by the FAO [14] as “protective and respectful of biodiversity and ecosystems; culturally acceptable, economically fair and accessible, nutritionally adequate, safe and healthy while optimizing natural and human resources” [13,15]. The World Health Organization (WHO) suggests that healthy and sustainable diets are rich in vegetables, fruits, and whole grains, with a limited intake of saturated fats, trans fats, sugar, and salt [13].

Research highlights that, although there is no formal definition for “healthy snacks”, dietary guidelines refer to snacks as highly nutritional, which ensures a “healthy life” and an “active lifestyle” [16]. It has been emphasized in different studies [8,17] that the definitions of “snack” and “healthy snack” are not clearly separated. Studies indicate that unhealthy snacks contain high amounts of fat and/or sugar [18,19,20], but the pleasure of consuming a food product is considered the most important determinant factor in food choices [8,21,22].

An increasing number of consumers and, consequently, producers are concerned about food products’ quality and safety. This has led to a rise in consumer demand for organic processed products that keep more of their original fresh plant characteristics [23]. One of these products is vegetable chips, which are seen as a healthier alternative to conventional chips [24].

In the context of the increasing consumption of organic products, the buying and consumption behavior has become a matter of interest for producers and market/retailers [25]. Moreover, consumers play a crucial role in promoting sustainable food systems associated with natural ingredients, free from flavor, color, and texture additives [3], influencing these systems through their choices and habits [13]. Previous studies on consumer choices regarding organic foods have predominantly focused on socio-demographic criteria, such as age, education, and household income [13,26,27,28,29]. For example, because of increased health consciousness, old-aged people reject intensely flavored snack foods, such as potato chips, which contain high amounts of salt, fat, and sugar [30], and choose healthier snacks like fruits [31]. Jánská (2020) [32] observed that 45% of single respondents reject organic food because of reasons related to the price. Many studies have shown that organic food consumption is positively influenced by increases in age, education, and income levels [33,34,35,36]. Moreover, people who live in urban areas are more open to consuming organic food [37,38,39]. Another important factor for organic food consumption is the existence of children within the household [40]. Women from Sweden, Indonesia, and Turkey have shown positive attitudes regarding organic products, as they are more preoccupied with health than men [41]. In Romania, based on research results of a study performed in Bucharest, the organic food market is influenced by socio-demographic factors, the most relevant being low income per capita [42]. In their study, Akter et al. (2023) [43] concluded that in the case of emerging countries, the small differences in the price and health properties between organic foods and traditional products have a significant effect on the level of organic food consumption.

Organic foods are no longer products that belong to specific groups of producers or consumers [44]. In developed countries, the organic products present in supermarkets are available in all major categories, such as fresh fruit and vegetables, meats, dry goods, bottled and tinned goods, dairy, baked goods, and confectionery [45].

Similar to worldwide trends, the Romanian food market is undergoing continuous changes as consumers increasingly prioritize health and environmental concerns [46]. They opt for organic food due to its reduced exposure to antibiotic-resistant bacteria and its reliance upon natural pesticide defense systems through plant phytochemicals [47]. Nevertheless, the Romanian organic fruits and vegetables market is still underdeveloped due to the small number of organic product consumers [41,48].

The expanding demand for healthy products with functional properties has prompted extensive research into the use of new ingredients rich in bioactive compounds, including nuts, fruits, fruit byproducts, vegetables, and vegetable byproducts [49]. Vegetables represent a major category of foods for rational and healthy diets due to active compounds and their nutritive values [50]. They can be consumed in various forms, either raw or fully or partially cooked. While fresh is often the best way to consume most vegetables, vegetable chips are gaining popularity as a snack. However, carrot, parsnip, beet, or sweet potato chips, prepared using the traditional frying method in a hot oil bath, are available on the market [51]. These vegetable chips, like conventional potato chips, are also unhealthy snacks due to their similar processing technology.

The Romanian market for organic vegetable chips, obtained through a gentle drying process, relies on imported products from countries such as Belgium, Holland, Ecuador, etc., and these chips can be purchased from specialized stores or ordered online.

To obtain vegetable chips that can be considered healthy snacks, the use of organic vegetables is crucial in the proper processing technique, such as drying and freeze-drying. These drying techniques offer several benefits, including extended shelf life, reduced microbial development, and decreased postharvest waste, resulting in significant savings in storage and transportation costs [52,53,54].

Given the current state of the art in the field, this study aims to contribute to the existing literature by providing insights into Romanian consumer preference towards vegetable chips, with a focus on organic vegetable chips vs. classic potato chips. The study is justified by the need to understand the Romanian consumers’ appetite for a healthy lifestyle and their choice of food products with high nutritional value.

## 2. Materials and Methods

### 2.1. Participants, Recruitment, and Procedure

To study Romanian consumers’ preference towards organic vegetable chips, an online survey was conducted based on a cross-sectional observational questionnaire [10,28,55,56]. The survey took place between November 2021 and July 2022 as an exploratory study, and the constituted sample of 1060 respondents was one of convenience, which means that its size was not previously calculated [57]. Respondents could access the questionnaire through platforms such as Facebook, LinkedIn, WhatsApp, and Yahoo. Prior to starting the survey, participants were informed about the purpose of the research and given the option to withdraw at any time.

The questionnaire was structured in two sections: the first section collected information relating to the socio-demographic characteristics of the respondents (e.g., gender, age, marital status, education level, household monthly net income). The second section focused on the consumer profile, including items related to the preference for organic vegetable chips. This item included ‘preference for organic vegetable chips vs. classic potato chips’, ‘consumption frequency of dried vegetables or products based on dried vegetables’, and ‘reason for replacing potato chips with a different type of organic vegetable chips’.

### 2.2. Study Objectives

The research employed a cross-sectional, descriptive, exploratory, and correlational design. To achieve the main objective of understanding Romanian consumers’ preferences for a healthy lifestyle by selecting food products with high nutritional value, particularly organic vegetable chips, the following specific objectives were formulated:

**O1.**  Identifying differences in socio-demographic factors (such as gender, age, marital status, education level, and household monthly net income) and their impact on the preference for organic vegetable chips (purple potatoes, carrot, parsnip, and beetroot) vs. classic potato chips.
**O2.**  Identifying the influence of (a) socio-demographic factors, (b) the consumption frequency of dried vegetables or products based on dried vegetables, and (c) the reason for replacing classic potatoes with the preferred chips (organic vegetable chips vs. classic potato chips).

### 2.3. Hypothesis Development and Research Procedures

As a confirmatory, descriptive, and exploratory research, the following questions were formulated:

**RQ1.**  Do socio-demographic variables differentiate chips preference (organic vegetable chips vs. classic potato chips) in Romania?
**RQ2.**  Is the chips preference (organic vegetable chips vs. classic potato chips) influenced by?

(a) Socio-demographic variables;
(b) The frequency of consuming dried vegetables or products based on dried vegetables;
(c) Reason for replacing potato chips with different types of organic vegetable chips (e.g., healthier, tastier, containing potato, only if they are found as a mix of several vegetables).

### 2.4. Hypothesis

Socio-demographic factors are important parameters for manufacturers and marketing departments. The reason behind testing these hypotheses was to identify the specific consumer segment that these departments should specifically address in the process of promoting organic vegetable chips.

To answer these questions, two general hypotheses were formulated, out of which hypothesis 1 had 5 specific hypotheses:

**H1.**  *It is expected that the socio-demographic variables will differentiate preferences for organic vegetable chips and classic potato chips in Romania.*

**H1.1.**  *More female consumers compared to males will prefer organic vegetable chips;*

**H1.2.**  *More older consumers compared to younger ones will prefer organic vegetable chips;*

**H1.3.**  *More married consumers than single ones will prefer organic vegetable chips;*

**H1.4.**  *More consumers with a higher education level compared to those with a lower education level will prefer organic vegetable chips;*

**H1.5.**  *More consumers with high incomes compared to those with low incomes will prefer organic vegetable chips.*

**H2.**  *It is expected that chips preference (organic vegetable chips vs. classic potato chips) in Romania (dependent variable-DV) will be influenced by the following independent variables (IV):*

(a) Gender, age, marital status, education level, household monthly net income;
(b) The consumption frequency of dried vegetables or products based on dried vegetables;
(c) The reason for replacing classic potato chips with different types of organic vegetable chips.

### 2.5. Data Analysis

The data were automatically recorded using Google Forms, imported to Microsoft Excel, and then to IBM SPSS 22, where processing and analysis were carried out. Frequency analysis, including percentage, was used for the qualitative data. Following the preliminary analysis, no missing data were reported.

The statistical procedures used to test the hypotheses were:

- Pearson’s chi-square test (Cramer’s V effect size) was used to identify if socio-demographic factors (gender, age, marital status, education level, and household monthly net income) differentiate chips preference. For gender, the Yates correction was applied due to the 2 × 2 table.
- Binary logistic regression was used to identify the influence of the independent variables (IV), including socio-demographic factors, frequency of consuming dried vegetables or products based on dried vegetables, and reasons for replacing potato chips with different types of organic vegetable chips on the dependent variable (DV), which represented chips preference. The dependent variable was a dummy variable, with participants who prefer classic potato chips defined as 0, and participants who prefer organic vegetable chips defined as 1.

The independent variables in the binary logistic regression were categorical:

(a) The gender variable had 2 categories: “male (1)” and “female (2)”;
(b) The age variable had 6 categories: “18–24 years (1)”, “25–34 years (2)”, “35–44 years (3)”, “45–54 years (4)”, “55–64 years (5)”, and “over 65 years (6)”;
(c) The marital status variable had 3 categories: “single (1)”, “in a relationship (2)”, and “married (3)”;
(d) The education level variable had 5 categories: “middle school education (1)”, “high school education (2)”, “post-secondary education (3)”, “university education (4), and “postgraduate education (5)”;
(e) The household monthly net income variable had 7 categories: “under 2500 lei (1)”, between “2501–3500 lei (2)”, “3501–4500 lei (3)”, “4501–5500 lei (4)”, “5501–6500 lei (5)”, “6501–8500 lei (6)”, and “over 8500 lei (7)”;
(f) The consumption frequency of dried vegetables or products based on the dried vegetables variable had 4 categories: „occasionally (1)”, “once a week (2)”, “3–4 times a week (3)”, and “daily (4)”;
(g) The reason for replacing classic potato chips with a different type of organic vegetable chips variable had 2 categories: “only if they are healthier (1)” and “other reasons (2)”.

The resulting model included the coefficients (β), their standard error (S.E.), the associated *p*-values (Sig.), the odds ratio (Exp(β)), and the confidence interval (CI for Exp(β)); Wald test; Hosmer–Lemeshow for goodness of fit for logistic regression models and Nagelkerke R^2^.

For both Pearson’s chi-square test and for binary logistic regression, the *p*-value was considered significant when *p* < 0.05 [58].

## 3. Results

### 3.1. Descriptive Statistics

The descriptive analysis was carried out to establish the respondent’s profile in relation to the studied variables, which included socio-demographic factors, preference for chips, the frequency of consuming dried vegetables or products based on dried vegetables, and the reason for replacing classic potato chips with a different type of organic vegetable chips.

Among the 1060 respondents, 699 (65.9%) reported that they prefer classic potato chips, 210 (19.8%) reported that they prefer organic vegetable chips, and 151 (14.2%) reported that they do not consume chips.

The analysis presented in this study focuses only on those 909 participants who consume chips, regardless of the type of chips consumed; the others were not included.

Of the 909 respondents, 699 (76.9%) prefer only classic potato chips, and 210 (23.1%) prefer organic vegetable chips. The prevalence of respondents who prefer classic potato chips can be observed in comparison to those who prefer organic vegetable chips.

Regarding the consumption frequency of dried vegetables or products based on dried vegetables: 649 (71.4%) consume occasionally, 135 (14.9%) once a week, 90 (9.9%) 3–4 times a week, and 35 (3.9%) daily. The prevalence of respondents who occasionally consume dried vegetables or products based on dried vegetables can be observed, along with the low percentage of daily consumers.

The reason for replacing classic potato chips with another variety was: “only if they are healthier” for 303 (33.3%) respondents, and other reasons for 606 (66.7%) respondents. The prevalence of respondents who replace classic potato chips with another variety for reasons other than health can be observed.

The sample structure of the 909 participants included in the study consisted of 288 (31.7%) males and 621 (68.3%) females, distributed as follows by age: 399 (43.9%) were between 18 and 24 years old, 203 (22.3%) were between 25 and 34 years old, 148 (16.3%) were between 35 and 44 years old, 115 (12.7 %) were between 45 and 54 years old, 33 (3.6 %) were between 55 and 64 years old, and 11 (1.2 %) were over 65 years old. In terms of marital status, the research participants have the following situation: 263 (28.9%) were single, 331 (36.4%) were in a relationship, and 315 (34.7%) were married.

In terms of education level, 1 (0.1%) had a middle school level education, 94 (10.3%) had a high school education, 23 (2.5%) had a post-secondary education, 569 (62.7%) had a university degree, and 222 (24.4%) were postgraduates.

Regarding household monthly net income categories, they were as follows: under 2500 RON (191, 21.0%), between 2501 and 3500 RON (163, 17.9%), between 3501 and 4500 RON (155, 17.1%), between 4501 and 5500 RON (92, 10.1 %), between 5501 and 6500 RON (84, 9.2%), between 6501 and 8500 RON (84, 9.2%), and over 8500 (140, 15.4%).

The respondent’s socio-demographic characteristics are presented in Table 1.

### 3.2. Inferential Statistics—Hypothesis Testing

To test the hypotheses regarding the differences in the distribution of respondents who prefer organic vegetable chips and those who prefer classic potato chips based on the socio-demographic characteristics, six analyses were run using the Pearson chi-square test.

The first specific hypothesis of H1 (H1.1. “More female consumers compared to males will prefer organic vegetable chips”) is not supported by the results (χ^2^(1) = 0.725, *p* = 0.394; Cramer’s V effect size = 0.031). The distribution of female and male respondents in relation to their preference for organic vegetable chips vs. classic potato chips is not statistically significant.

The contingency table, 2 (preference for chips) × 2 (gender), shows that: (i) among male consumers, 21.2% prefer organic vegetable chips compared to 78.8% who prefer classic potato chips; (ii) among female consumers, 24.0% prefer organic vegetable chips compared to 76.0% who prefer classic potato chips (Table 2).

The second specific hypothesis of H1 (H1.2. “More older consumers compared to young ones will prefer organic vegetable chips”) is supported by the obtained results. The association between age and preference for organic vegetable chips vs. classic potato chips is statistically significant (χ^2^(5) = 36.670 and *p* < 0.001, Cramer’s V effect size = 0.201). The contingency table, 2 (preference for chips) × 6 (age ranges), shows the following: (i) among consumers aged 18–24 years, 14.3% prefer organic vegetable chips compared to 85.7% who prefer classic potato chips; (ii) among consumers aged 25–34 years, 28.6% prefer organic vegetable chips compared to 71.4% who prefer classic potato chips; (iii) among consumers aged 35–44 years, 29.7% prefer organic vegetable chips compared to 70.3% who prefer classic potato chips; (iv) among consumers aged 45–54 years, 29.6% prefer organic vegetable chips compared to 70.4% who prefer classic potato chips; (v) among consumers aged 55–64 years, 45.5% prefer organic vegetable chips compared to 54.5% who prefer classic potato chips; and (vi) among consumers over 65 years old, 18.2% prefer organic vegetable chips compared to 81.8% who prefer classic potato chips. The prevalence of preference for classic potato chips is observed in all age categories, but it is most noticeable in those aged between 18 and 24 years and those over 65 years old. Moreover, the preference for organic vegetable chips increases with respondent age, except for those over 65 years old (Table 3).

The third specific hypothesis of H1 (H1.3. “More married consumers than single ones will prefer organic vegetable chips”) is supported by the results. The association between marital status and preference for organic vegetable chips vs. classic potato chips is statistically significant (χ^2^(2) = 10.329 and *p* = 0.006, Cramer’s V effect size = 0.107). The contingency table, 2 (preference for chips) × 3 (marital status), shows the following: (i) among single consumers, 17.9% prefer organic vegetable chips compared to 82.1% who prefer classic potato chips; (ii) among consumers in a relationship, 21.8% prefer organic vegetable chips compared to 78.2% who prefer classic potato chips; (iii) among married consumers, 28.9% prefer organic vegetable chips compared to 71.1% who prefer classic potato chips. The prevalence of preference for classic potato chips is observed in all categories of respondents, but it is most pronounced among those who are single. An increased preference for organic vegetable chips can be observed when consumers are married (Table 4).

The fourth specific hypothesis of H1 (H1.4. “More consumers with a higher educational level compared to those with a lower education level will prefer organic vegetable chips”) is supported by the results. The association between the preference for organic vegetable chips vs. classic potato chips and educational level is statistically significant (χ^2^(4) = 36.046 and *p* < 0.001, Cramer’s V effect size = 0.199). The contingency table, 2 (preference for chips) × 5 (educational level), shows the following: (i) there is only one consumer with a middle school level of education, and they prefer organic vegetable chips; (ii) among consumers with a high school education, 12.8% prefer organic vegetable chips compared to 87.2% who prefer classic potato chips; (iii) among consumers with post-secondary education, 13.0% prefer organic vegetable chips compared to 87.0% who prefer classic potato chips; (iv) among consumers with a university education, 19.9% prefer organic vegetable chips compared to 80.1% who prefer classic potato chips; and (v) among consumers with postgraduate education, 36.5% prefer organic vegetable chips compared to 63.5% who prefer classic potato chips. The prevalence of the preference for classic potato chips is observed in all categories but is pronounced among respondents with high school and post-high school education. Among respondents with university and postgraduate education, the preference for organic vegetable chips is increased, indicating that consumer preference is influenced by education level (Table 5).

The fifth specific hypothesis of H1 (H1.5. “More consumers with high incomes compared to those with low incomes will prefer organic vegetable chips”) is supported by the results. The association between the preference for organic vegetable chips vs. classic potato chips and household income is statistically significant, indicated by the coefficient χ^2^(6) = 22.018 and *p* = 0.001, Cramer’s V effect size = 0.156. The contingency table, 2 (preference for chips) × 7 (household monthly net income), shows the following: (i) among consumers with an income below 2500 RON, 14.1% organic vegetable chips compared to 85.9% who prefer classic potato chips; (ii) among consumers with an income between 2501 and 3500 RON, 18.4% organic vegetable chips compared to 81.6% prefer who classic potato chips; (iii) among consumers with an income between 3501 and 4500 RON, 23.2% prefer organic vegetable chips compared to 76.8% who prefer classic potato chips; (iv) among consumers with an income between 4501 and 5500 RON, 27.2% prefer organic vegetable chips compared to 72.8% who prefer classic potato chips; (v) among consumers with an income between 5501 and 6500 RON, 29.8% prefer organic vegetable chips compared to 70.2% who prefer classic potato chips; (vi) among consumers with an income between 6501 and 8500 RON, 35.7% prefer organic vegetable chips compared to 64.3% who prefer classic potato chips; and (vii) among consumers with an income over 8500, 26.4% prefer organic vegetable chips compared to 73.6% who prefer classic potato chips. The preference for classic potato chips is prevalent in all income categories, but it is most present among those with an income below 2500 and those between 2501 and 3500 RON. It can be observed that a higher household income influences the consumer preference regarding organic vegetable chips (Table 6).

To answer the second research question (RQ2), we formulated the general hypothesis H2. To test this hypothesis, a binary logistic regression analysis was conducted, with chip preference (organic vegetable chips vs. classic potato chips) as the dependent variable (DV) and the following independent variables (IV): gender, age, marital status, education level, household monthly net income, the frequency of consuming dried vegetables or products based on dried vegetables, and the reason for replacing classic potato chips with a different type of organic vegetable chips.

The result of the binary logistic regression analysis, indicated by χ^2^ = 102,906 with 22 degrees of freedom and a sample size of 909 (N = 909), demonstrates statistical significance at a *p*-value of less than 0.001. This implies that the independent variables (IVs) significantly influence the variation observed in the dependent variable (DV). The Nagelkerke R² index is 0.162, suggesting that the entire model explains 16.2% of the variation in the results. The *p*-value in the Hosmer and Lemeshow test was 0.949 (χ^2^ = 2.747, df = 8), indicating that there is no difference between the observed and predicted model (*p* > 0.05). A non-significant Chi-square indicates that the data fit the model well.

It was observed that only education level (high school education, university education) and frequency of consumption of dried vegetables or products based on dried vegetables (occasionally) have a statistical impact on the preference for chips (DV) (Wald = from 5.988 to 15.957; *p* < 0.05).

The frequency of consumption of dried vegetables or products based on dried vegetables (occasionally) has the highest statistically significant impact on the preference for organic vegetable chips (Wald = 15.957, *p* < 0.001), while education level (university education) has the weakest statistically significant impact (Wald = 5.988, *p* = 0.014). Meanwhile, the other categories have no significant impact on preference for organic vegetable chips (Sig. > 0.05).

Table 7 presents the model results, including coefficients (β), standard errors (S.E.), Wald test, associated *p*-values (Sig.), odds ratio (Exp(β)), and confidence interval (CI for Exp(β)).

## 4. Discussion

As anticipated at the beginning of the study, the logistic model results indicate that certain categories of the analyzed variables significantly impact the preference for organic chips: “the frequency of consumption of dried vegetable products” (occasionally) and “education level” (high school education, university education).

We expected gender to be one of the variables influencing the preference for organic vegetable chips; however, the coefficient for gender in the binary regression model was not significant. We started with the premise that females are often more interested in health and nutrition, especially organic vegetables, based on data from previous studies [41]. For example, in a study focusing on organic food consumption, it was found that 94.5% of women and 80.5% of men prefer organic vegetables, considering them beneficial for their health [59]. Another study indicates that actual buyers of organic products are primarily females [60]. In our study, the proportion of women consuming organic chips was higher than that of men; however, this difference is not statistically significant and may be attributed to chance (results for H1.1.). It is possible that both women and men equally pay attention to their diet, as it is well known that poor dietary habits can lead to various digestive and other health issues. Considering the results of the binary regression and those obtained from hypothesis testing (if gender differentiates the preference for chips), we can conclude that the consumer’s gender (male or female) is not a significant factor in the marketing sector.

Social status is another variable for which the results of the binary regression model indicate that it does not influence the preference for organic vegetable chips, as the coefficient’s significance is not statistically meaningful (for none of the categories of the variable). In hypothesis testing for the differentiation of chips preference (organic vegetable chips vs. classic potato chips), the result was statistically significant. In the case of “married” consumers, the observed frequency was higher than the theoretical frequency, and the percentage difference between the observed and theoretical frequencies was the highest compared to what was determined for the “single” and “in a relationship” categories. For both “single” and “in a relationship” respondents, the observed frequency was lower than the theoretical frequency. The percentage difference (between observed and theoretical frequencies) decreases from “single” to “in a relationship”, suggesting that “in a relationship” respondents have a stronger preference for organic vegetable chips compared to “single” respondents. The obtained results align with data presented in another study, which states that women with children represent a factor in the profile of actual consumers of organic products [60].

The results obtained from hypothesis testing for the other socio-demographic factors can play an important role in promoting organic vegetable chips to different consumer segments, considering factors such as age, educational level, and monthly income. We formulate this statement based on the significant statistical differences identified between the categories of each listed socio-demographic factor.

For example, in the case of hypothesis testing aimed at identifying age-related differences in preference for chips (organic vegetable chips vs. classic potato chips), the results were statistically significant. For the age categories “18–24 years” and “above 65 years”, the observed frequency was lower compared to the theoretical frequency, indicating that in these groups, the preference for organic vegetable chips might be lower than expected. For the other age categories, the situation is different; the observed frequencies are higher than the theoretical frequency, suggesting that in these age groups, the preference for organic vegetable chips is higher than theoretically anticipated. Considering the percentage differences (between observed and theoretical frequencies) for each age category and the situations where observed frequencies were higher or lower compared to theoretical frequencies, we can infer that the results indicate a fluctuation in the preference for organic vegetable chips. Additionally, we can deduce that respondents in the “55–64 years” age group exhibit the highest preference for organic vegetable chips compared to any other age category. In the binary regression model, the only category for which the coefficient had a positive value was “55–64 years.” We can state that respondents in this category might have a slight preference for organic vegetable chips. However, the result is not significant enough to draw firm conclusions.

For the education level variable, the coefficient value from the binary logistic regression is statistically significant for two of the categories. For example, in the case of “high school education”, the significance level of the coefficient B indicates significant differences between this category and the reference category “postgraduate education”. The negative coefficient value suggests that respondents with a „high school education” are less likely to prefer organic vegetable chips compared to those with a „postgraduate education”. Similarly, for the „university education”, we can observe significant differences between this category and the reference category („postgraduate education”), supported by the statistically significant coefficient value. Again, the negative coefficient value suggests that respondents with „university education” are less likely to prefer organic vegetable chips compared to those with „postgraduate education”.

The differentiation in chips preference (organic vegetable vs. classic potato) based on the educational level variable was statistically significant. When comparing observed frequencies to theoretical frequencies for the category of consumers of organic vegetable chips, a trend emerges: as the educational level increases, the preference for organic vegetable chips also increases. This is evident in the decreasing percentage differences between observed and theoretical frequencies as educational levels rise, particularly when observed frequencies are lower than theoretical ones. In the case of respondents in the “postgraduate education” category, the observed frequency exceeded the theoretical frequency.

Price is an important factor in the consumer purchasing decisions, as they often evaluate the price-to-quality ratio. In this sense, we can identify two categories of consumers: a category willing to pay more for organic vegetable chips or higher quality potato chips and those who can opt for more affordable options. Consumers who choose organic vegetable chips are more interested in using natural or organic ingredients in their food. This interest may stem from the fact that organic vegetable chips are obtained from raw materials that come from organic agriculture; hence, they are perceived as more environmentally friendly.

Considering these aspects, we found it relevant to identify how net income differentiates the preference for organic vegetable chips vs. classic potato chips, and our hypothesis is statistically supported. Taking into account the percentage differences (between observed and theoretical frequencies) corresponding to each “net income” category and considering situations where observed frequencies were lower or higher than theoretically estimated frequencies, we can conclude that respondents with a higher net income have a stronger inclination towards preferring organic vegetable chips.

The results of the binary regression model indicate that none of the coefficients of the net income categories are statistically significant. For the income categories: “4501–5500 lei”, “5501–6500 lei”, and “6501–8500 lei”, the positive coefficients’ values suggest that respondents in these net income categories could have a slight preference for organic vegetable chips. However, the result is not statistically significant enough to assert this as a conclusive finding. Our study’s findings are in line with some previous studies [61] and contradictory to others [26] that identified relationships between net income and the choice to consume organic vegetables.

In the binary regression model, the variable “frequency of consumption of dried vegetables and products based on dried vegetables” had the highest Wald value, and the coefficient value was statistically significant for the “occasionally” category. The negative coefficient value suggests that respondents who consume dried vegetables and products based on dried vegetables “occasionally” are less likely to prefer organic vegetable chips compared to those who consume them “daily”.

We expected that the “reason for replacing potato chips with a different type of organic vegetable chips”, specifically the category “only if they are healthier”, would influence the preference for organic vegetable chips. Some studies suggest that a significant share of consumers frequently consider organic products as healthier and tastier, valuing them for their higher vitamin and mineral content [26]. However, in the binary logistic regression model, the coefficient for this category was not statistically significant, indicating no significant differences between respondents who would choose organic vegetable chips because they are healthier and those in the reference category (“other reasons”).

Consumer preferences are important aspects of consumer behavior. The understanding of factors influencing consumer behavior is currently a frequently studied topic [25]. In the field of psychology, understanding the underlying needs that drive consumption or purchasing decisions [62] and analyzing these factors to anticipate consumer behavior [63] can be valuable assets for the marketing sector. This understanding can benefit the marketing area by enabling market segmentation and the identification of specific groups with similar needs and preferences. In organic product marketing, special attention should be paid to local economic and social conditions to meet consumer needs [64]. Taking all these into account, it becomes evident that consumers’ personal preferences can be regarded as important factors for producers and marketing departments [65].

Regardless of the perspective, the study of consumers’ preferences for organic vegetable chips can undoubtedly bring benefits to producers, traders, and consumers alike.

Since personal preferences can be influenced by family health consciousness, food safety consciousness, perceiving vegetables as healthy, and enjoying vegetables, the study results can be a valuable source of information for understanding attitudes towards vegetables in the TPB model.

## 5. Conclusions

The research design was conceived from the perspective of shaping the profile of organic vegetable chips consumer. In this study, we explored potential predictive factors regarding the preference for organic vegetable chips. Regarding chips preference, three categories were identified within the sample: respondents who prefer classic potato chips, respondents who prefer organic vegetable chips, and a third category who do not prefer chips. The latter category was excluded from the sample that constituted the database for this study. Within the analyzed sample, approximately a quarter of the respondents indicated a preference for organic vegetable chips.

The hypotheses aimed at identifying differences in preference between organic vegetable chips and classic potato chips based on the socio-demographic characteristics of Romanian consumers. These hypotheses were statistically significant for age, marital status, education level, and net monthly income. The exception is represented by the hypothesis regarding gender differences in the preference for organic vegetable chips vs. classic potato chips.

The conclusions obtained from the SPSS 22 analysis (binary logistic regression) indicate that, regarding education level, respondents who fall into the “high school education” and “university education” categories are less likely to prefer organic vegetable chips compared to those in the “postgraduate education” category (reference category).

Another conclusion drawn from the same analysis highlights that individuals who consume dried vegetables or products based on dried vegetables “occasionally” have fewer chances of preferring organic vegetable chips compared to those who consume dried vegetables or related products “daily”.

The obtained results in this study provide valuable insights into the factors that influence the preference for organic vegetable chips in Romania. This information can be useful for both chip manufacturers and retailers in developing effective strategies to promote the consumption of healthy snacks. These insights can enhance the awareness of chip producers regarding consumption preference patterns for snacks in Romania, which is a relatively less-explored niche. This study is among the few that enable a better understanding of Romanian preferences, and these preferences represent important factors that encourage the consumption of healthy snacks.

## Figures and Tables

**Table 1:** The sample structure.

Characteristics	Share in the Sample	Percentage (%)
Gender	Female	68.3
	Male	31.7
Age (years)	18–24	43.9
	25–34	22.3
	35–44	16.3
	45–54	12.7
	55–64	3.6
	over 65	1.2
Marital status	Single	28.9
	In a relationship	36.4
	Married	34.7
	Middle school education	0.1
Education level	High school education	10.3
	Post-secondary education	2.5
	University education	62.7
	Postgraduate education	24.4
Household monthly net income (RON)	under 2500	21.0
	2501–3500	17.9
	3501–4500	17.1
	4501–5500	10.1
	5501–6500	9.2
	6501–8500	9.2
	over 8500	15.4

**Table 2:** The distribution of participants based on their preference for chips in relation to gender.

			Preference for Chips		Total Lines
			Organic Vegetable Chips	Classic Potato Chips	
Gender	Male	Count	61	227	288
		Expected count	66.5	221.5	288
		% within a *	21.2	78.8	100
		% within b *	29.0	32.5	31.7
	Female	Count	149	472	621
		Expected count	143.5	477.5	621
		% within a	24.0	76.0	100
		% within b	71.0	67.5	68.3
	Total columns	Count	210	699	909
		Expected count	210	699	909
		% within a	23.1	76.9	100
		% within b	100	100	100

* a—Gender; b—Preference for chips.

**Table 3:** The distribution of participants based on their preference for chips in relation to age.

			Preference for Chips		Total Lines
			Organic Vegetable Chips	Classic Potato Chips	
Age	18–24	Count	57	342	399
		Expected count	92.2	306.8	399
		% within a *	14.3	85.7	100
		% within b *	27.1	48.9	43.9
	25–34	Count	58	145	203
		Expected count	46.9	156.1	203
		% within a	28.6	71.4	100
		% within b	27.6	20.7	22.3
	35–44	Count	44	104	148
		Expected count	34.2	113.8	148
		% within a	29.7	70.3	100
		% within b	21.0	14.9	16.3
	45–54	Count	34	81	115
		Expected count	26.6	88.4	115
		% within a	29.6	70.4	100
		% within b	16.2	11.6	12.7
	55–64	Count Expected count % within a % within b	15	18	33
			7.6	25.4	33
			45.5	54.5	100
			7.1	2.6	3.6
	Over 65	Count Expected count % within a % within b	2	9	11
			2.5	8.5	11
			18.2	81.8	100
			1.0	1.3	1.2
	Total columns	Count	210	699	909
		Expected count	210	699	909
		% within a	23.1	76.9	100
		% within b	100	100	100

* a—Age; b—Preference for chips.

**Table 4:** The distribution of participants based on their preference for chips in relation to marital status.

			Preference for Chips		Total Lines
			Organic Vegetable Chips	Classic Potato Chips	
Marital status	Single	Count	47	216	263
		Expected count	60.8	202.2	263
		% within a *	17.9	82.1	100
		% within b *	22.4	30.9	28.9
	In a relationship	Count	72	259	331
		Expected count	76.5	254.5	331
		% within a	21.8	78.2	100
		% within b	34.3	37.1	36.4
	Married	Count	91	224	315
		Expected count	72.8	242.2	315
		% within a	28.9	71.1	100
		% within b	43.3	32.0	34.7
	Total columns	Count	210	699	909
		Expected count	210	699	909
		% within a	23.1	76.9	100
		% within b	100	100	100

* a—Marital status; b—Preference for chips.

**Table 5:** The distribution of participants based on their preference for chips in relation to education level.

			Preference for Chips		Total Lines
			Organic Vegetable Chips	Classic Potato Chips	
Education level	Middle school level	Count	1	0	1
		Expected count	0.2	0.8	1
		% within a *	100	0.0	100
		% within b *	0.5	0.0	0.1
	High school education	Count	12	82	94
		Expected count	21.7	72.3	94
		% within a	12.8	87.2	100
		% within b	5.7	11.7	10.3
	Post-high school education	Count	3	20	23
		Expected count	5.3	17.7	23
		% within a	13.0	87.0	100
		% within b	1.04	2.9	2.5
	University education	Count	113	456	569
		Expected count	131.5	437.5	569
		% within a	19.9	80.1	100
		% within b	53.8	65.2	62.7
	Postgraduate education	Count Expected count % within a % within b	81	141	222
			51.3	170.7	222
			36.5	63.5	100
			38.6	20.2	24.4
	Total columns	Count	210	699	909
		Expected count	210	699	909
		% within a	23.1	76.9	100
		% within b	100	100	100

* a—Education level; b—Preference for chips.

**Table 6:** The distribution of participants based on their preference for chips in relation to income.

			Preference for Chips		Total Lines
			Organic Vegetable Chips	Classic Potato Chips	
Household monthly net income (RON)	under 2500	Count	27	164	191
		Expected count	44.1	146.9	191
		% within a *	14.1	85.9	100
		% within b *	12.9	23.5	21.0
	2501–3500	Count	30	133	163
		Expected count	37.7	125.3	163
		% within a	18.4	81.6	100
		% within b	14.3	19.0	17.9
	3501–4500	Count	36	119	155
		Expected count	35.8	119.2	155
		% within a	23.2	76.8	100
		% within b	17.1	17.0	17.1
	4501–5500	Count	25	67	92
		Expected count	21.3	70.7	92
		% within a	27.2	72.8	100
		% within b	11.9	9.6	10.1
	5501–6500	Count	25	59	84
		Expected count	19.4	64.6	84
		% within a	29.8	70.2	100
		% within b	11.9	8.4	9.2
	6501–8500	Count	30	54	84
		Expected count	19.4	64.6	84
		% within a	35.7	64.3	100
		% within b	14.3	7.7	9.2
	over 8500	Count	37	103	140
		Expected count	32.3	107.7	140
		% within a	26.4	73.6	100
		% within b	17.6	14.7	15.4
	Total columns	Count	210	699	909
		Expected count	210	699	909
		% within a	23.1	76.9	100
		% within b	100	100	100

* a—Household monthly net income (RON); b—Preference for chips.

**Table 7:** Predicting chips preference based on socio-demographic variables, the frequency of consumption of dried vegetables or products based on dried vegetables (Q_2_), and the reason for replacing classic potato chips with a different type of organic vegetable chips (Q_3_).

	B	S.E.	Wald	df	Sig.	Exp(B)	95% C.I. for EXP(B)	
							Lower	Upper
GENDER								
male	−0.163	0.189	0.744	1	0.388	1.177	0.813	1.705
AGE								
18–24 years	−0.849	0.888	0.913	1	0.339	0.428	0.075	2.440
25–34 years	−0.197	0.886	0.049	1	0.824	0.821	0.145	4.662
35–44 years	−0.089	0.887	0.010	1	0.920	0.915	0.161	5.207
45–54 years	−0.212	0.897	0.056	1	0.813	0.809	0.139	4.695
55–64 years	0.603	0.948	0.404	1	0.525	1.827	0.285	11.708
MARITAL STATUS								
single	0.106	0.264	0.162	1	0.687	1.112	0.663	1.865
in a relationship	0.298	0.247	1.464	1	0.226	1.348	0.831	2.185
EDUCATION LEVEL								
middle school education (only one respondent)	22.786	40,192.969	0.000	1	1.000	7,866,208,019.034	0.000	0.000
high school education	−1.070	0.370	8.372	1	0.004	0.343	0.166	0.708
post-secondary education	−1.134	0.681	2.776	1	0.096	0.322	0.085	1.221
university education	−0.497	0.203	5.988	1	0.014	0.608	0.408	0.906
INCOME								
under 2500 lei	−0.392	0.328	1.425	1	0.233	0.676	0.355	1.286
2501–3500 lei	−0.282	0.305	0.852	1	0.356	0.754	0.415	1.372
3501–4500 lei	−0.036	0.292	0.015	1	0.901	0.964	0.544	1.710
4501–5500 lei	0.153	0.322	0.227	1	0.634	1.166	0.620	2.190
5501–6500 lei	0.196	0.329	0.353	1	0.552	1.216	0.638	2.317
6501–8500 lei	0.528	0.317	2.780	1	0.095	1.696	0.911	3.157
Q2								
occasionally	−1.039	0.260	15.957	1	<0.001	0.354	0.212	0.589
once a week	0.073	0.304	0.058	1	0.810	1.076	0.593	1.953
3–4 times a week	−0.076	0.450	0.028	1	0.866	0.927	0.383	2.241
Q3								
only if they are healthier	−0.087	0.181	0.234	1	0.629	0.916	0.643	1.306
Constant	0.110	0.934	0.014	1	0.907	1.116		

## Data Availability

The data used to support the findings of this study can be made available by the main author upon request.
